# Semi-quantitative assessment of diffuse hepatic uptake seen in I-131 scans – an indicator of functioning thyroid tissue and disease burden in differentiated thyroid cancer

**DOI:** 10.1186/s13044-019-0065-1

**Published:** 2019-04-25

**Authors:** Prasanta K. Pradhan, Suruchi Jain, Madhusudhanan Ponnuswamy, Amitabh Arya, Manish Ora

**Affiliations:** 1 SGPGIMS, Lucknow, India; 2 AIIMS, Bhopal, India; 3 JIPMER, Puducherry, India

**Keywords:** Differentiated thyroid cancer, Liver uptake, Thyroglobulin, Hepatic to thigh ratios

## Abstract

**Background:**

To semi-quantitatively analyze liver uptake of I-131 in diagnostic and post-therapy scans by calculating hepatic to thigh ratios (HTR) and evaluate its clinical significance in management of differentiated thyroid cancer.

**Method:**

Two hundred forty-nine patients were included in the study. Hepatic to thigh ratio (HTR) of counts were calculated for 249 diagnostic and 104 post-therapy scans. Patients were divided into six study groups based on their disease status:1-Serum thyroglobulin (serum Tg) negative (serum Tg ≤ 4 ng/dl) and scan negative; 2-Thyroid remnant only; 3-Thyroid remnant and lymph node metastasis; 4- Tg positive (serum Tg > 4 ng/dl) and scan negative; 5-Bone or/and lung metastasis, and 6-Only lymph node metastasis. Comparison of HTR between these groups was done using one-way ANOVA test. Correlation of HTR with serum Tg, serum thyroid stimulating hormone (TSH), anti-thyroglobulin antibody (ATg) titer and therapeutic dose of I-131 was also assessed.

**Results:**

Comparison of HTR between different study groups (1 to 6) showed significant difference in HTR (*p* = .001). Study group 5 (bone or/and lung metastasis) showed significantly higher mean HTR compared to other groups (*p* = 0.001). There was only a weak correlation between serum Tg and HTR (r = 0.395). Dose of I-131 administered also had a weak correlation with HTR (r = 0.207).

**Conclusion:**

HTR has good correlation with functional status of tumor cells, while it has weak correlation to therapeutic dose of I-131 administered and serum Tg. Increased HTR predicts significant disease burden in the form of distant bone and lung metastasis and may potentially be used as a second prognostic factor apart from serum Tg.

## Introduction

Diagnostic Iodine-131 (I-131) scans are done routinely to evaluate remnant in the thyroid bed, regional lymph node and distant metastases prior to deciding dose of I-131 therapy in differentiation thyroid cancer [1]. Liver is visualized physiologically in patients with remnant thyroid tissue and residual or metastatic thyroid cancer tissue [2]. Diffuse hepatic visualization in the I-131scans has been a topic of research for a long time. In previous studies liver visualization has been attributed to metabolism of hormones and thyroglobulin synthesized by differentiated thyroid cancer cells [3–5]. The clinical significance of the hepatic uptake of I-131 in management of differentiated thyroid cancer remains uncertain. In this retrospective study, we have semi-quantitatively analyzed visualization of I-131 in liver in diagnostic and post-therapy scans by calculating hepatic to thigh ratios (HTR) to test the difference in liver visualization in patients with different disease status.

## Material and methods

### Study population

Data from 280 consecutive patients, who visited thyroid clinic for differentiated thyroid cancer over a period of 1 year, was retrospectively analysed. 249 patients, who fulfilled the inclusion criteria, were included in the study. Inclusion criteria were:

1. Normal liver function tests- serum glutamic oxaloacetic transaminase, serum alanine amino-transferase, serum alkaline phosphatase, and serum bilirubin.
2. Availability of data on: serum thyroglobulin (Tg), serum antithyroglobin antibody (ATg), serum TSH and amount of I-131 administered.

All the 249 patients included in the study had their diagnostic scans and 104 of these patients also had their post-therapy scans.

### Study groups

Patients were divided into six groups based on their I-131 scan findings and serum Tg levels:

1. Disease free patients (I-131 scan negative and serum Tg negative (Tg ≤ 4 ng/dl)).
2. Patients with thyroid remnant only.
3. Patients with thyroid remnant and lymph node metastasis.
4. Patients with whole body radioiodine (WBRI) scan negative and serum Tg positive (Tg > 4 ng/dl).
5. Patients with bone or/and lung metastasis only.
6. Patients with lymph node metastasis only and no thyroid remnant.

Whole body diagnostic scans were acquired 48 h after oral administration of 5 mCi of I-131. All patients had been prepared by undergoing 4 weeks of thyroid hormone withdrawal. Post-therapy scan timing varied between 2 and 5 days post administration of I-131 depending on the timing of discharge of the patient based on patient’s radiation level. WBRI scans were done on dual head gamma camera (Infinia Hawkeye 4, GE healthcare) with medium energy general purpose (MEGP) collimator. WBRI scans were acquired in both anterior and posterior projections. In case of any interpretative difficulty regarding metastatic lesion in planar I-131 image, SPECT/CT was done. Region of Interest (ROIs) were drawn in the region of liver and mid-thigh and average counts were noted. Ratio of average counts per pixel in the liver ROI and mid-thigh ROI was calculated and recorded as hepatic to thigh ratio (HTR). The hepatic to thigh ratio is meant to correct hepatic uptake for background counts. Anterior images of diagnostic I-131 scans were used for comparison of HTR in between different study groups and correlating HTR with serum Tg, serum ATg and serum TSH. Anterior images of post-therapy scans were used for correlating HTR with dose of I-131 administered. Serum Tg, serum TSH and serum ATg of all the patients were recorded. Serum Tg was measured by Fully Automated Chemiluminescence Immuno Assay. Serum TSH was measured by Ultra-Sensitive Chemiluminescence Assay. Serum ATg levels were measured using Solid Phase Enzyme-Immuno Assay. Serum ATg values < 225 IU/mL were considered negative, values between 225 IU/mL and 320 IU/mL were considered equivocal and values > 320 IU/mL were considered as positive.

### Statistical analysis

Paired sample t-test was applied for comparison of HTR obtained from anterior image and that obtained from posterior image. Comparison of HTRs of diagnostic and post-therapy scans was made by paired t-test. Pearson correlation was calculated to study the correlation of HTR with dose of I-131 administered, serum Tg, serum ATg and serum TSH. Comparison of HTR value among different study groups was done using One-way ANOVA test (Post Hoc: Student-Newman-Keuls test).

## Results

Total number of males was 75 (30%) and total number of females was 174 (70%). Mean age of patients was 37.84 years (range: 8–77 years). Distribution of patients based on histopathological diagnosis is given in Table 1.

**Table 1:** Classification based on histopathological report

Histopathology	No. of patients	Percentage (%)
PCT	208	83.53
Classical PCT	195	78.33
Follicular variant	11	4.41
Tall cell variant	2	0.8
FCT	41	16.47
Widely invasive	38	15.26
Minimally invasive	2	0.8
Hurthle cell variant	1	0.4

*PCT* papillary carcinoma of thyroid, *FCT* follicular carcinoma of thyroid

100 patients (40.2%) were disease free [WBRI scan negative and serum Tg negative (Tg ≤ 4 ng/dL)]. 28 patients had only remnant tissue in the neck and had no evidence of metastasis. 18 patients had thyroid remnant and lymph node metastasis but no evidence of skeletal or lung metastasis. 62 patients had negative WBRI scan but positive serumTg (Tg > 4 ng/dl). 27 patients had only bone or/and lung metastasis and no evidence of remnant thyroid tissue or lymph node metastasis. 14 patients had only lymph node metastasis with no apparent remnant thyroid tissue. Detailed classification along with mean HTR values in different study groups can be seen in Table 2.

**Table 2:** Number of patients in different study groups and mean HTR

Group No.	Patients	Number	Mean HTR ± S.D.
1.	Disease free patients [WBRI scan negative and serum thyroglobulin negative (Tg ≤ 4 ng/dL)]	100	2.35 ± 0.507
2.	Patients with thyroid remnant only	28	2.65 ± 0.675
3.	Patients with thyroid remnant and lymph node metastasis	18	3.07 ± 0.688
4.	Patients with WBRI scan negative and serum thyroglobulin positive (Tg > 4 ng/dL)	62	2.31 ± 0.407
5	Patients with bone or/and lung metastasis only	27	4.62 ± 2.843
6.	Patients with lymph node metastasis only and no remnant	14	2.12 ± 0.442

*WBRI* whole body radioiodine

### Comparison of HTR obtained from anterior image and that obtained from posterior image

No significant difference was found in mean HTR obtained from anterior (2.64 ± 1.20) and posterior (2.59 ± 1.56) images of diagnostic 131-I scan (*p* = 0.246) and showed a good correlation (r = 0.910) (Table 3). Similarly, mean HTR obtained from anterior (3.70 ± 1.97) and posterior (3.62 ± 2.31) images of post therapy scan showed no significant difference (*p* = 0.253). Moreover, there was excellent correlation (r = 0.949).

**Table 3:** A. Comparison of HTR obtained from anterior image and that obtained from posterior image B. Comparison of HTR obtained from diagnostic scan and that obtained from post-therapy scan

A.				
	Anterior image	Posterior image	*P* value	Correlation(r)
Mean HTR(obtained from Diagnostic scans)	2.64 ± 1.20	2.59 ± 1.56	0.246	0.910
Mean HTR(obtained from Post-therapy scans)	3.70 ± 1.97	3.62 ± 2.31	0.283	0.949
B.				
	Diagnostic scans	Post-therapy scans	P value	Correlation(r)
Mean HTR(obtained from Anterior image)	2.64 ± 1.20	3.70 ± 1.97	0.001	0.797
Mean HTR(obtained from Posterior image)	2.59 ± 1.56	3.62 ± 2.31	0.001	0.855

### Comparison of HTR obtained from diagnostic scan and that obtained from post-therapy scan

On comparing mean HTR obtained from anterior images of diagnostic I-131 scan (2.64 ± 1.20) with mean HTR obtained from anterior images of post-therapy I-131 scan (3.70 ± 1.97), paired sample t-test showed a significant difference in mean HTR (*p* = 0.001) but there was good correlation between them (r = 0.797). Similarly, paired sample t-test showed a significant difference in mean HTR (p = 0.001) obtained from posterior images of diagnostic I-131 scan (2.59 ± 1.56) with posterior images of post-therapy I-131 scan (3.62 ± 2.31), with a good correlation (r = 0.855).

### Correlation of HTR with dose of radioiodine administered for therapeutic purpose

104 patients underwent high dose I-131 therapy/ablation with mean dose of 60.0 mCi (dose range 30–200 mCi). There was only a weak correlation of post-treatment HTR with therapeutic dose of I-131 administered (r = 0.207).

### Comparison of HTR value among different study groups

**Fig. 1:**
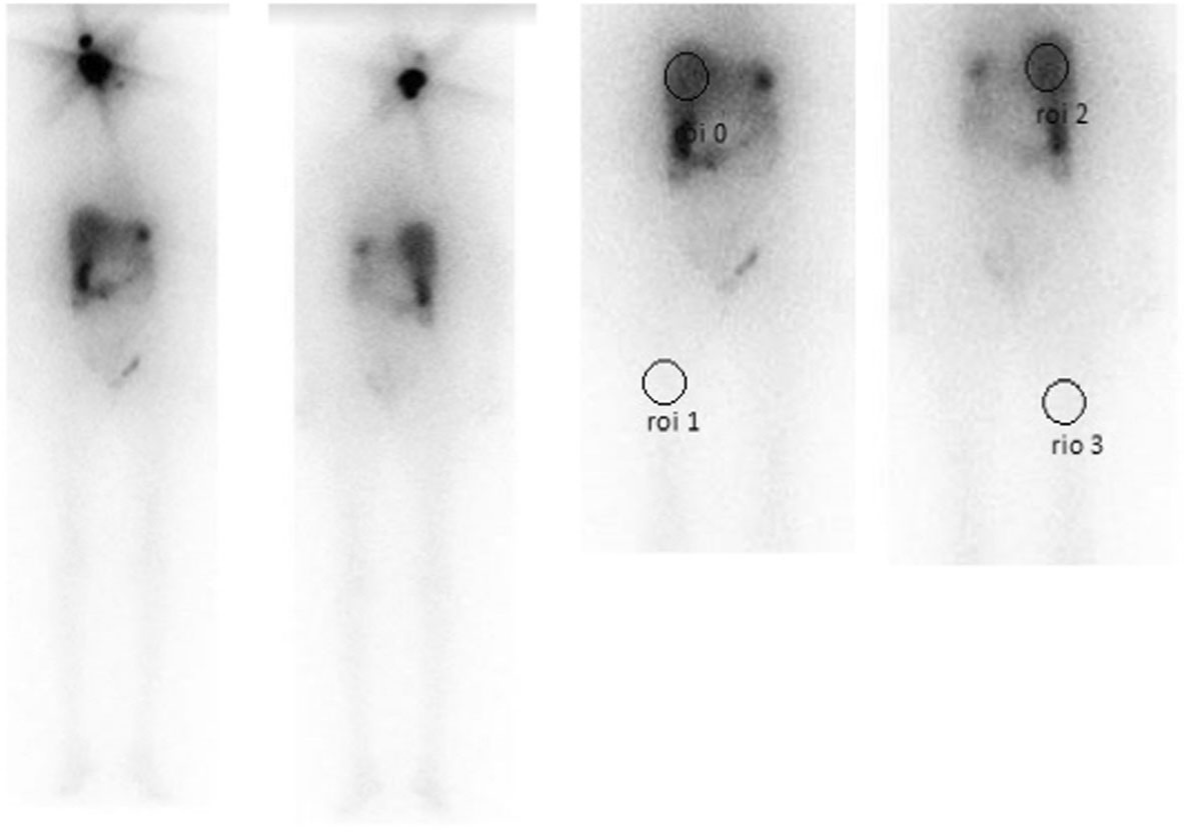
A patient with multiple skeletal metastasis and significant liver uptake on Whole body radioiodine scan

**Fig. 2:**
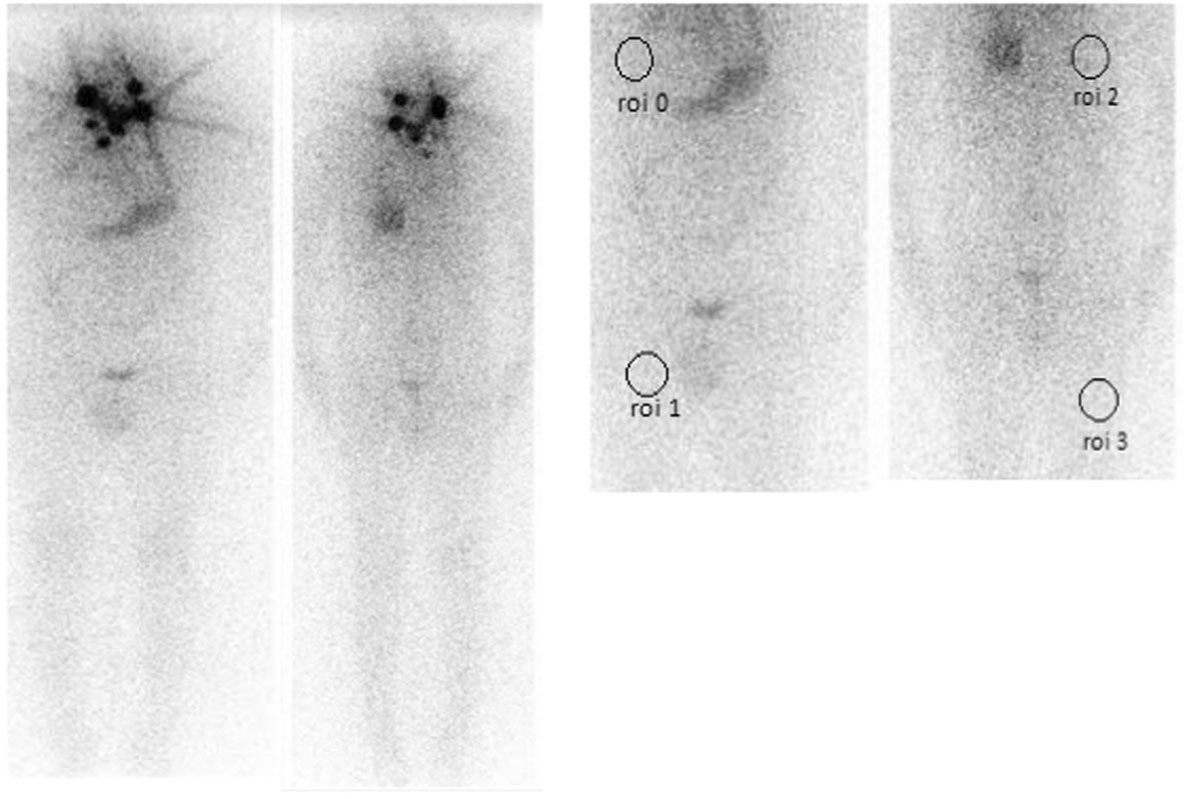
A patient with significant lymph node metastasis and insignificant liver uptake on Whole body radioiodine scan

Comparison of HTR between different study groups (1 to 6) showed significant difference in HTR (*p* = .001). Value of HTR in different study groups is shown in Table 2. There was no significant difference (*p* = 0.233) in HTR between patients who had negative WBRI scan but raised serum Tg (Group 4) and patients who were disease free (Group 1). Mean HTR was found to be highest in patients who have bone or/and lung metastasis (Group 5) and lowest in patients who have only lymph node metastasis or negative radioiodine scan (Group 1, 4 and 6). Mean HTR values were intermediate in patients who had only thyroid remnant (Group 2). Example of a patient with significant liver uptake and multiple skeletal metastases is depicted in Fig. 1. On the other hand, Fig. 2 depicts a patient with significant lymph node metastasis but insignificant liver uptake.

There was also significant difference in HTR in patients with papillary thyroid carcinoma (PTC) as compared with follicular thyroid carcinoma (FTC) (*p* = 0.001). Mean HTR obtained from patients with FTC was 4.91 ± 2.78 while that of patients with PTC was 3.26 ± 1.38.

### Correlation of HTR with serum Tg

Mean serum Tg of patients was 64.18 ng/dl (range: 0.20–300.0 ng/dl). There was only a weak positive correlation (r = 0.395) between serum Tg and HTR.

### Correlation of HTR with serum ATg

Mean serum ATg level was 112.28 IU/mL (S.D. ±156.69). Serum ATg values were negative in 264 (94.28%) patients, equivocal in 4 (1.4%) patients while positive in 12 (4.28%) patients. There was only a very weak correlation of serum ATg with HTR (r = 0.101).

### Correlation of HTR with serum TSH

Mean serum TSH values was 106.00mIU/L (S.D. ±50.69). There was negative correlation (r = − 0.254) between HTR and serum TSH. The patients with serum Tg ≥ 300 ng/dL were separately analyzed and it was found that the negative correlation between HTR and serum TSH was stronger in these patients (r = − 0.557).

## Discussion

Hepatic I-131 uptake has been noted in diagnostic and post radioiodine therapy scans done for thyroid cancer [5]. The exact mechanism for visualization of liver is still uncertain [6–9].

Some studies [5, 10, 11] suggest that radio-iodinated thyroglobulin released from functioning cancer tissue is metabolized in liver and results in visualization of liver. According to this theory, liver visualization should correlate with serum Tg.

However, some authors believe that when I-131 is administered for diagnostic scan or for therapy, it is secreted by remnant thyroid tissue as ^131^I labeled thyroid hormones, predominantly radio labeled thyroxine [12].Thyroid hormones metabolized in liver are excreted in the bile and partially hydrolyzed in the bowel. As liver is the centre of metabolism and excretion of these hormones, liver is visualized on radioiodine scans, diagnostic or post-therapy [12].

Other authors [13–15] argue that nonspecific iodinated protein identified in the serum from patients with thyroid cancer may be responsible for liver visualization. There is evidence implicating certain peroxidases such as myeloperoxidase and lactoperoxidase that organify iodine in nonspecific iodoproteins such as albumin. Iodoproteins produced by these enzymes are secreted by the stomach, salivary glands, thymus and can accumulate in the liver [13].

HTR was obtained from both anterior and posterior images of all the patients in this study. There was no significant difference between HTR obtained from anterior and posterior images (*p* = 0.246), suggesting that HTR can be obtained from either anterior or posterior image alone without any effect on this value. Quantitative evaluation of hepatic uptake has been done only by one of the previous studies [16] by Michihiro et al. but they used cranial uptake as background uptake. In our study thigh uptake was taken as background because bone metastasis in appendicular skeleton are rare in contrast to metastasis in the head and neck,which could result in higher uptake in a background ROI overlying the cranium due to scatter. In post-therapy scans, we found only a weak correlation between HTR and the therapeutic dose of radioiodine administered (r = 0.207). This is in contrast to a few previous studies [9, 14], in which visual grading of liver uptake showed correlation with the therapeutic dose of radioiodine administered. This difference may be because visual grading is a subjective tool whereas in this study an objective parameter (HTR) is used, which is likely to be more precise.

While some studies have shown significant correlation of hepatic uptake with serum Tg level (*p* < 0.01) [5, 10, 11], we found that HTR correlates only weakly with serum Tg(r = 0.395). This is similar to the findings of Ozgur Omur et al.*,* who showed no significant correlation between the serum Tg level and hepatic uptake score (*P =* 0.58, *r =* 0.020) [14].

The patients with serum Tg ≥ 300 ng/dl were separately analyzed and it was found that HTR has negative correlation with serum TSH (r = − 0.557). It is likely that larger functional remnant tissue synthesizes more hormones, results in an enhanced metabolism of hormones in liver and therefore, a higher HTR. Higher hormone levels do not allow serum TSH to rise as high as in patients with smaller functional thyroid tissue and a lower synthesized hormone level.

Another study by Ziessman *et al* [9] supports the above finding, and show a positive correlation of diffuse hepatic uptake and serum ^131^I-protein bound iodine (r = 0.494). The administered ^131^I-iodide becomes concentrated in the functioning thyroid cells, where it is ultimately metabolized into thyroxine (T_4_). And this is known as “protein-bound iodine” (PBI), since the T_4_ is predominantly bound to thyroxine-binding globulin [17].

While a few studies [5, 18] have shown that diffuse hepatic uptake correlates with presence of residual thyroid tissue or DTC metastasis, none of them have assessed its significance with respect to type of metastasis. In this study, a significant finding is that liver uptake and thereby, HTR is highest and significantly different (*p* < 0.05) in patients who have bone or/and lung metastasis (mean = 4.62). On the other hand, patients who have only lymph node metastasis have lower values of HTR (mean = 2.12). This implies that tumors spreading hematogenously are more likely to retain hormone synthesis ability than those tumors that spread through the lymphatic route. This observation suggests that, uptake in the liver is also related to the type of metastasis, which in turn is related to the histopathology of the tumor (papillary or follicular cancer). Higher sensitivity of WBRI scan in cases of FTC as compared with cases of PTC might also be due to the same reason that FTC are more functional in comparison to PCT [19]. Finding of this study, that HTR is significantly higher (*p* ≤ 0.05) in patients with FTC compared with those of PTC also support the same hypothesis.

In this study, patients who have negative WBRI scan but raised serum Tg have no significant difference in HTR in comparison to patients who are disease free (defined as negative WBRI scan along with serum Tg < 4 ng/dl) (*P* = 0.233). This suggests that liver uptake is identical in patients who have negative scan irrespective of their serum Tg level. These findings suggest that hormone production, which is the inherent part of tumor differentiation, is responsible for visualization of liver in the scan. In contrast, whole body radioiodine scan negative and serum Tg positive tumors lose their ability to concentrate iodine and synthesize hormones during de-differentiation. Therefore, visualization of liver and hence HTR is related with hormone synthesis ability of the tumor and not related with serum Tg levels alone. This observation is contrary to previous studies [5, 10].

In patients with skeletal metastasis, it has often been observed that if serum Tg of patients is very high (≥300 ng/dl), these patients have suppressed TSH even after withdrawal of thyroid hormone indicating functioning thyroid tissue. But in patients with lymph nodal metastasis or WBRI scan negative patients with very high serum Tg, serum TSH has been found to rise sufficiently after 4 weeks of l-thyroxine withdrawal. Hence, production of thyroglobulin and thyroid hormones appear to be two independent characteristics of differentiated thyroid cancer and probably are not interrelated. Visualization of liver and higher value of HTR are likely to be related to hormone production by the tumor and hence, is not affected by raised serum Tg alone. In patients with raised serum Tg, if HTR is also raised, it suggests that patient has significant metastatic functioning thyroid tissue and would benefit by I-131 administration in follow up whereas patients with raised serum Tg on initial scan and no definite increased HTR are more likely to be scan negative in follow up scans and hence will not require repeated radio-iodine administration. This idea is supported by Michihiro et al.*,* who showed that diffuse hepatic uptake predicts disease related progression [16].

### Limitations of the study

Although liver ROI was drawn over the right lobe of liver in all the scans which is representative of I-131 uptake by the liver but still there is a possibility of subjective error depending on the variability of site of ROI placement.

## Conclusion

HTR has good correlation with functional status of tumor cells in differentiated thyroid cancer, while it is independent of total dose of radioiodine administered and serum Tg. Increased HTR predicts significant disease burden in the form of distant bone and lung metastasis and potentially may be used as a second prognostic factor apart from serum Tg. Potential use of HTR as a prognostic factor in addition to serum Tg needs to be verified by further follow up studies.

## Abbreviations

ATg: Anti thyroglobulin antibody
HTR: Hepatic to thigh ratio
I-131: Iodine 131
ROI: Region of Interest
Tg: Thyroglobulin
TSH: Thyroid Stimulating Hormone
WBRI: Whole body radio-iodine
